# Off-label use of repurposed ivermectin for SARS-CoV-2 infection should be banned by authorities unless efficacy is proven

**DOI:** 10.51866/lte.664

**Published:** 2024-07-17

**Authors:** Josef Finsterer

**Affiliations:** 1 MD, PhD, Neurology and Neurophysiology Center, Postfach 20, Vienna, Austria. Email: fifigs1@yahoo.de

**Keywords:** SARS-CoV-2 infection, Ivermectin, Side effects, Questionnaire, Cross-sectional design

**Dear editor,**

We read with interest the article by Filza Nur Athirah et al. on a cross-sectional study of the knowledge and attitude towards and frequency of ivermectin use for SARS-CoV-2 infection (SC2I) among 391 adults evaluated using an electronic, self-administered, anonymous, structured and validated questionnaire distributed via Google Forms.^1^ The authors found that 3.6% of the included respondents were taking ivermectin but had moderate knowledge and attitude towards its use. It was concluded that Malaysians have moderate knowledge and attitude towards ivermectin and only rarely use it. The study is impressive, but some points require discussion.

The first point relates to the design of the study. Electronic questionnaires have several disadvantages. First, it is not easy to determine whether the answers given are true. Second, it cannot be determined whether respondents are the ones answering the questions and not someone else. Third, respondents’ ability to answer the questions correctly and appropriately cannot be easily verified. Fourth, additional questions that arise after the questionnaire is completed cannot be asked any longer.

The second point is that the conclusions drawn do not seem to be supported by the data provided.^1^ The study was conducted at a single centre and included only 391 respondents. It is therefore not representative of the entire population, which is why the general conclusions about the entire population of Malaysia are not justified. A multicentre design that includes respondents from all over the country would be desirable to achieve the aim of the study.

The third point is that, according to the methods section, the questionnaire has been validated before use in the current study.^1^ It is important to know what type of respondents was surveyed and whether this test group was compared to a control group. Further, the results of the validation, whether they have been published and whether they are accessible must be clarified.

The fourth point is that the study was conducted from March to May 2022, suggesting that at least some of the patients included had received SARS-CoV-2 vaccination. Therefore, how many of the enrolled patients were fully vaccinated at the time of the study and whether there was a difference in the results between the vaccinated and unvaccinated respondents must be explained.

The fifth point is that the use of ivermectin can be complicated not only by side effects such as allergies, dizziness, vomiting, seizures, coma and death^1^ but also by myalgia; muscle stiffness; arthralgia; swollen and tender lymph nodes; swelling of the face, arms, hands, lower legs and feet; chest pain; chills; cold sweats; coughing; eye or eyelid irritation; arrhythmias; fever; dysuria; sore throat; weight gain; tarry stool; increased sleepiness or tiredness; agitation; back pain; blurry vision; disorientation; headache; urinary incontinence; hallucinations; mood change; unusual dullness or change in mental status.^2,3^ Whether any of the included respondents reported such side effects must be specified.

The sixth point is that the inclusion criteria were not detailed. In particular, it must be explained why students (n=277) predominated the sample. The predominance of students could represent a selection bias that may strongly influence the results.

In summary, the excellent study has limitations that should be addressed before drawing finalconclusions. Clarifying the weaknesses would strengthen the conclusions and could improve thestudy. Before a repurposed drug for SC2I treatment is used, it is advisable to test the drug for itseffectiveness and side effects. The off-label use of repurposed drugs is understandable given theemergency of the pandemic but should be permitted by health authorities only in the context ofcontrolled studies and after confirmation of the drugs’ effectiveness.

## References

[1] Filza Nur Athirah T, Aina Amanina AJ, Nor Safwan Hadi NA (2024). Knowledge, attitude and prevalence of ivermectin use as coronavirus disease treatment: a crosssectional study among a Malaysian population.. Malays Fam Physician..

[2] Drugs.com. (2024). Ivermectin side effects. Know more be sure..

[3] Kaur B, Blavo C, Parmar MS (2024). Ivermectin: a multifaceted drug with a potential beyond anti-parasitic therapy.. Cureus..

